# Generation of a dataset for DoW attack detection in serverless architectures

**DOI:** 10.1016/j.dib.2023.109921

**Published:** 2023-12-05

**Authors:** José Manuel Ortega Candel, Francisco José Mora Gimeno, Higinio Mora Mora

**Affiliations:** Department of Computer Science and Technology, Alicante University, Alicante, Spain

**Keywords:** Functions as a service, Serverless, Denial of wallet, Botnet

## Abstract

Denial of Wallet (DoW) attacks refers to a type of cyberattack that aims to exploit and exhaust the financial resources of an organization by triggering excessive costs or charges within their cloud or serverless computing environment. These attacks are particularly relevant in the context of serverless architectures due to characteristics like pay-as-you-go model, auto-scaling, limited control and cost amplification.

Serverless computing, often referred to as Function-as-a-Service (FaaS), is a cloud computing model that allows developers to build and run applications without the need to manage traditional server infrastructure. Serverless architectures have gained popularity in cloud computing due to their flexibility and ability to scale automatically based on demand. These architectures are based on executing functions without the need to manage the underlying infrastructure. However, the lack of realistic and representative datasets that simulate function invocations in serverless environments has been a challenge for research and development of solutions in this field.

The aim is to create a dataset for simulating function invocations in serverless architectures, that is a valuable practice for ensuring the reliability, efficiency, and security of serverless applications. Furthermore, we propose a methodology for the generation of the dataset, which involves the generation of synthetic data from traffic generated on cloud platforms and the identification of the main characteristics of function invocations. These characteristics include SubmitTime, Invocation Delay, Response Delay, Function Duration, Active Functions at Request, Active Functions at Response.

By generating this dataset, we expect to facilitate the detection of Denial of Wallet (DoW) attacks using machine learning techniques and neural networks. In this way, this dataset available in Mendeley data repository could provide other researchers and developers with a dataset to test and evaluate machine learning algorithms or use other techniques based on the detection of attacks and anomalies in serverless environments.

Specifications Table

**Table:** 

Subject:	*Computer Science* *Cryptography and Cybersecurity*
Specific subject area:	The specific subject area of this topic is focused on creating a dataset that can be used to simulate and study Denial of Wallet (DoW) attacks in serverless computing architectures.
Data format:	Raw, Analyzed, Filtered
Type of data:	*Function invocations in a serverless architecture in CSV format* Table, Chart, Graph, Figure
Data collection:	This dataset represents function invocations in serverless environments and can be used to train and evaluate machine learning and deep learning algorithms for the detection of these types of attacks.
Data source location:	This dataset has been generated from other public datasets and tools with the aim of having a starting data set to investigate the Denial Of wallet threat. The primary source for the generation of this dataset is DoWTS (Denial-of-Wallet Test Simulator) available in the following GitHub repository: https://github.com/psykodan/DoWTS
Data accessibility:	Data accessibility Repository name: Mendeley Data Data identification number: 10.17632/g8g9vdxyvn.1 Direct URL to data: https://data.mendeley.com/datasets/g8g9vdxyvn/1

## 1 Value of the Data

• The dataset is available in the following public Mendeley data repository and is accessible without restrictions: https://data.mendeley.com/datasets/g8g9vdxyvn/1
• This dataset could be useful in the detection of Denial of Wallet (DoW) attacks in many industries and applications, including IoT (Internet of Things), E- commerce, Finance and Fintech.
• The use of this dataset can significantly improve security in serverless architectures in several ways like anomaly detection, threat detection and mitigation.
• This dataset may be relevant to work related to the detection of possible DoW attacks on serverless architectures, as it records various parameters related to function calls, as well as other performance metrics such as memory and CPU usage.
• Analysis of the data would help to identify the parameters necessary to identify possible DoW attacks, mainly due to the variability of events and the number of functions invocations at specific window times. Between these parameters we can highlight the number of active functions and delay times at request and response.
• The dataset can be used to train and test machine learning models, such as anomaly detection algorithms or predictive models. These models can recognize patterns and behaviors associated with DoW attacks, providing early threat detection. Previous work on anomaly detection in cybersecurity, such as studies on machine and deep learning for Denial-of-Service attacks, can serve as a reference for model development [1,2].
• This dataset is intended to be trained by supervised algorithms such as Decision Tree where the bot variable is what determines whether an attack has occurred. Other neural network models such as multilayer perceptron or recurrent networks could also be used for Denial-of-Service and Denial-of-Wallet attacks detection [3,4].
• The data could be used as a reference for predicting DoW attacks. The dataset can be used to test and validate the accuracy of predictive models.

## 2 Data Description

A dataset that predicts Denial of Wallet (DoW) attacks has various potential use cases and applications like early threat detection, cost management in cloud platforms and resource allocation optimization.

Researchers and security practitioners could use this dataset to develop predictive models that identify emerging DoW attacks. This can lead to early threat detection and a more proactive response to mitigate potential financial and operational risks.

The dataset generated includes function invocations with the event that occurred, response times, duration of functions, functions activated in request and response, memory and CPU usage.

The dataset contains invocations to 50 different functions over a 24-hour period and each function provides the event that triggered it. To give more relevance to the types of events, the dataset contains a column called **functionTrigger** which contains the following possible events:

**Table:** 

{"http", "storage", "sql", "stream", "notification", "email","nosql"}

In Table 1 we can see data related with transactions generated in the dataset.

**Table 1:** Data set transactions.

Total transactions	187087
Attack transactions (bot=1)	131072
Legitimate transactions(bot=0)	56015
Percentage of attack transactions	70.06 %
Percentage of legitimate transactions	29.94 %

Table 2 shows the name of each column together with the data type:

**Table 2:** Data set columns and data types.

Column	Data Type
Id	int64
IP	object
bot	bool
FunctionId	int64
functionTrigger	object
timestamp	object
SubmitTime	int64
RTT	int64
InvocationDelay	float64
ResponseDelay	float64
FunctionDuration	float64
ActiveFunctionsAtRequest	int64
ActiveFunctionsAtResponse	int64
maxcpu	float64
avgcpu	float64
p95maxcpu	float64
vmcategory	object
vmcorecountbucket	int64
vmmemorybucket	float64

With the aim of obtaining the most relevant metrics to be added to the dataset, we have used other studies that analyse the main metrics for measuring the performance of workloads on serverless infrastructure [5], [6], [7], [8]. Among the main metrics analysed we can highlight:

• **Communications performance**. A serverless application is usually composed of several functions that interact with each other and with other cloud services. There are different models of function interaction to initiate communication between them, such as the use of a function orchestrator or the use of the cloud provider's SDK.
• **Start-up latency to execute the function**. Because serverless functions are started on demand, the processing latency of each request contains the start-up latency. In addition, because the execution unit is typically small and of short duration (milliseconds), serverless computing can be more sensitive to spikes in overhead.
• **Execution time**. In serverless architectures we use execution time to know which functions require a higher computational cost, and hardware-level performance metrics are used to obtain the instructions executed.
• **CPU usage**. This measures the ratio between the time the application spends on the CPU, both in user and kernel space. This metric helps to detect applications that use resources more than those initially allocated.
• **Memory usage.** The detection of memory spikes is important to determine if the configuration is correct, as well as the billing of applications. It also allows providers to limit the number of containers allocated to deploy the application.
• **Input/Output operations**. The average throughput of file system and network I/O operations decreases with the number of function calls.

## 3 Experimental Design, Materials and Methods

Since our goal is to create a new dataset, we start by analyzing what other data sources we have available within the serverless ecosystem that allows us to have a base from which to start. The test dataset has been generated from the following tools and datasets:

• **Dataset generated by the DoW simulator** whose tool can be found in the GitHub repository [9]. This tool allows generating synthetic data of normal or malicious traffic as part of a botnet and could be used in the training of algorithms for DoW detection. This tool simulates the traffic generated in the requests of a serverless application and internally it generates thousands of requests per second and, therefore, could be used to simulate DDoS/DoW attacks [10,11]. It has the capacity to calculate the cost of function invocations by simulating the traffic that functions can generate. It also generates usage log data for each call with a label denoting whether the traffic is part of a bot or legitimate. This data could be used in future research to differentiate legitimate traffic from botnet-generated traffic [8]. The purpose of this tool is the generation of datasets that contain function invocations on different cloud platforms such as AWS Lambda, IBM functions, Google Functions and Azure Functions and analyze if these invocations are botnet origin for DoW detection research.
• **Concurrent execution monte carlo serverless functions** across aws, google, ibm and alibaba is a dataset that contains function invocations in different cloud providers [13]. The purpose of this dataset is to simulate the execution of serverless functions in cloud providers.
• **Microsoft Azure dataset.** The Microsoft Azure dataset is a collection of 52,000 roles that were invoked 8.8 billion times over a 2-week period. The three main objects in the dataset are roles, applications and users, which are identified by anonymous hash identifiers. This data is divided into three main parts: a time series of calls, execution times and memory usage [14,15]. The purpose of this dataset is to identify the main metrics we can use in serverless environments to measure function invocations, execution times and memory usage.
  ○ **Function invocations:** The time series containing the number of calls of a function in each minute of the 14 days of testing.
  ○ **Execution times**: The dataset contains the average execution time and a fixed set of percentiles for each function.
  ○ **Memory usage**: The dataset includes the average memory usage for each application and is also broken down into a fixed set of percentiles. Unlike the other data that is recorded per function, memory usage is recorded for the entire application because Azure aggregates resource allocation for functions that belong to the same application.

### 3.1 Methodology

Regarding the methodology we have used to generate synthetic data from serverless function invocations, we could highlight the following steps:

• **Define the data format:** Determine the structure and fields of the serverless function invocation data. For example, you could include fields such as function name, arguments passed, function response, execution time, etc.
• **Configure events:** In a serverless architecture, functions are triggered in response to specific events. You can configure different events to trigger function execution. Examples of events include changes to a message queue, updates to a database, or HTTP requests.
• **Generate events and function invocations**: For this task, we could use tools such as the DoW test simulator [9,12]. This tool allows us to configure an event generator that continuously generates synthetic traffic based on patterns for different cloud platforms such as AWS, Google Cloud, IBM and Azure.
• **Store the data generated**: To facilitate analysis, the results are stored in a csv file.
• **Validate and analyze the generated data**: Finally, we perform a validation of the generated data to ensure that the requirements are consistent.
• **Clean and prepare the dataset:** Once the serverless function invocation data has been generated, it would be important to clean and prepare the dataset. This phase may include removing duplicate data, handling null values, or performing specific transformations depending on the needs of further analysis.

The following steps have been taken to generate this serverless function call dataset.

• **Generate an initial dataset with the Dow test simulator tool** [9,12]. This initial dataset contains information related to IP addresses and function identifiers, along with the indicator if that invocation is part of a botnet. We also generate a column called **functionTrigger** that contains the event that triggered the function invocation. Table 3 shows the columns generated in the first step:

**Table 3:** Dataset columns generated in the first step.

Id	IP	bot	FunctionId	functionTrigger	timestamp

• **Using the dataset with concurrent executions on different cloud platforms**[13]: In this phase the objective is to add information related to submit time (submitTime), that is the duration in milliseconds (ms) that a network request takes to go from a point of a source to a destination and back to the starting point (RTT), request delay time (InvocationDelay), response delay time (ResponseDelay), function duration (FunctionDuration), active functions in the request (ActiveFunctionsAtRequest) and active functions in the response (ActiveFunctionsAtResponse). In this step we would have a dataset composed with the columns of the previous step plus the columns in Table 4:

**Table 4:** Dataset columns generated in the second step.

SubmitTime	RTT	Invocation Delay	Response Delay	Function Duration	ActiveFunctions AtRequest	ActiveFunctions AtResponse

• **Using the dataset that contains information related to the type of virtual machine, CPU and memory usage in the Azure cloud platform** [14,15]: In this case the objective is to add information related to the maximum CPU usage (maxcpu), average CPU usage (avgcpu), maximum CPU usage 95% percentile (p95maxcpu), virtual machine category (vmcategory), virtual machine cores (vmcorecountbucket) and virtual machine memory (vmmemorybucket). In this step we would have a dataset composed with the columns of the previous steps plus the columns in Table 5:

**Table 5:** Dataset columns generated in the final step.

maxcpu	avgcpu	p95maxcpu	vmcategory	vmcorecountbucket	vmmemorybucket

**Fig. 1:**
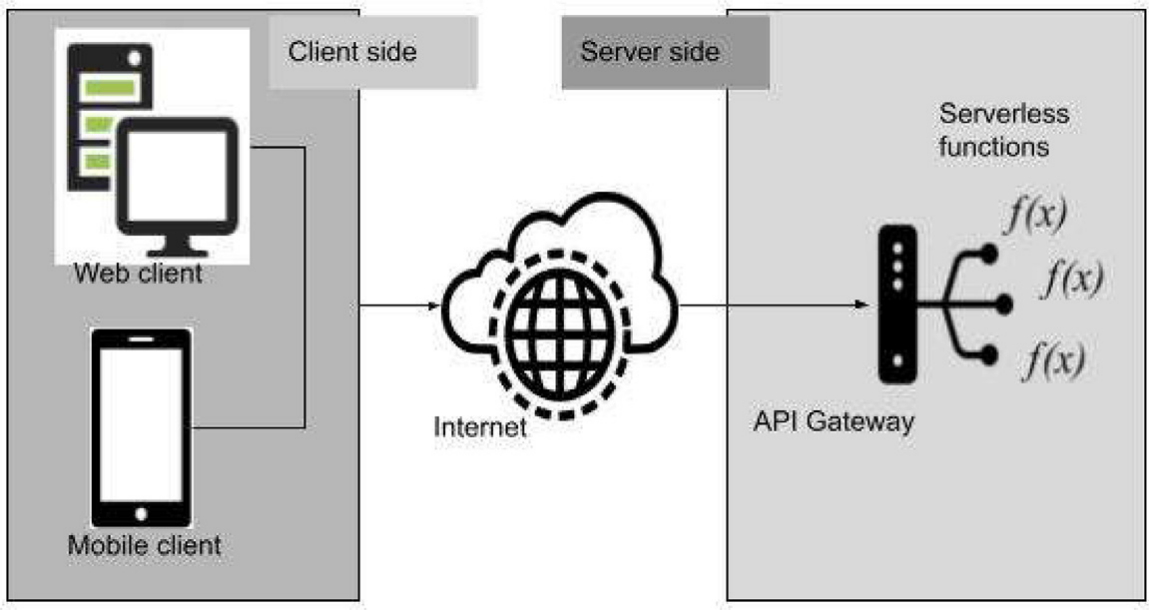
Serverless application architecture.

Fig. 1 represents the typical architecture of a serverless application where the client side make requests to an API Gateway that will be the one that invokes the different functions depending on users demand.

**Fig. 2:**
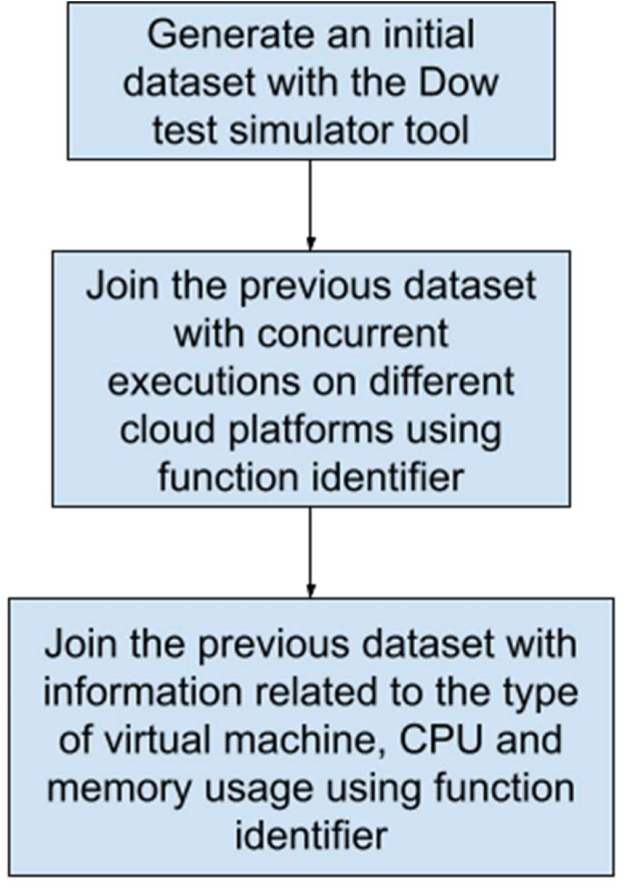
Steps for dataset generation.

Figure 2 summarizes the dataset generation process in 3 steps:

These are the meanings of the variables introduced in our dataset:

• **IP**: Represents the IP address from which the request is made.
• **bot**: Represents a Boolean variable if the request originates from a botnet.
• **FunctionId**: Represents the identifier of the serverless function.
• **functionTrigger**: Represents the event or action that triggers the execution of the serverless function.
• **timestamp**: Represents the moment at which the invocation of the serverless function occurs.
• **submitTime**: Represents refers to the moment when the request or event is initiated or submitted to the serverless platform. It marks the point in time when the serverless function is triggered to execute in response to an event, such as an HTTP request, a message in a queue, or a change in a database.
• **RTT (Round-Trip Time):** Represents a measure of the time it takes for a request to be sent from a client to a server and for the response to be received back from the server to the client.
• **InvocationDelay**: Represents the time it takes from when an event triggers the execution of a serverless function to when the function starts running and processing the event.
• **ResponseDelay**: Represents the amount of time it takes for the serverless function to produce a response or complete its execution after being invoked.
• **FunctionDuration**: Represents the amount of time it takes for a serverless function to execute and complete its task.
• **ResponseDelay**: Represents the amount of time it takes for the serverless function to produce a response or complete its execution after being invoked.
• **ActiveFunctionsAtRequest**: Represents the number of functions that are active in the request during a serverless invocation.
• **ActiveFunctionsAtRequest**: Represents the number of functions that are active in the response during a serverless invocation.
• **vmcategory**: Represents the virtual machine category.
• **vmcorecountbucket**: Represents the number of cores of virtual machine.
• **vmcategory**: Represents the virtual machine category.

### 3.2 Most representative graphs

**Fig. 3:**
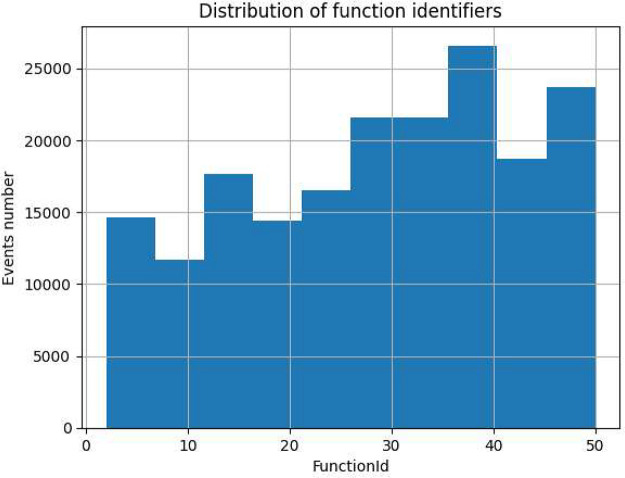
Distribution of function identifiers.

We could start by representing the histogram of the most representative variables. Fig. 3 represents a distribution of function identifiers (50 functions) on the x-axis together with the events generated by each function identifier on the y-axis.

**Fig. 4:**
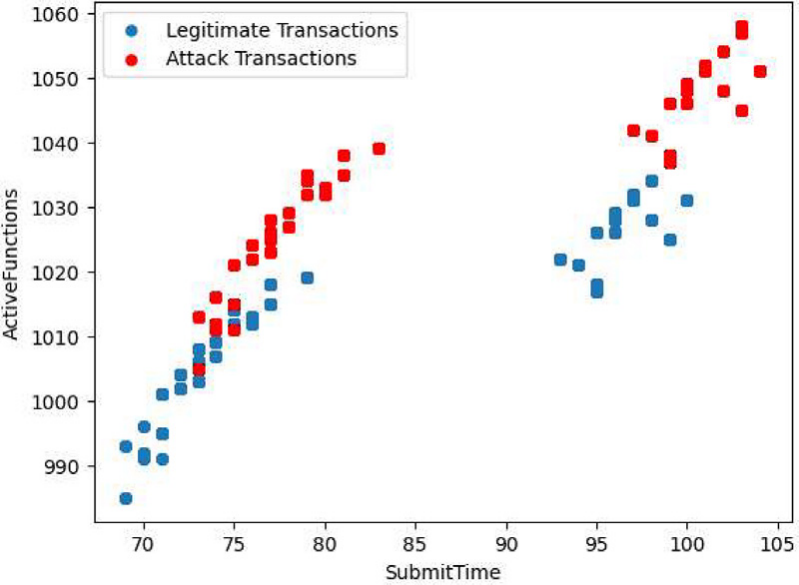
SubmitTime vs ActiveFunctions.

As illustrated in Fig. 4, we can see the relationship between the variable that represents the time to submit the function (**SubmitTime**) and the number of active functions (**ActiveFunctions**). We can see that for attack transactions both variables are increased, and we could separate the types of transactions using these variables.

In the Table 6 we see the statistics of attack and legitimate transactions considering the **ActiveFunctions** column. As we can see in the table, the dataset consists of 56015 legitimate transactions (bot=0) and 131072 attack transactions (bot=1).

**Table 6:** Statistics of attack and legitimate transactions.

	Legitimate transactions	Attack transactions
count	56015.000000	131072.000000
mean	1011.179916	1042.442177
std	10.046044	15.000330
min	975.000000	1015.000000
25 %	1005.000000	1032.000000
50 %	1011.000000	1043.000000
75 %	1019.000000	1056.000000
max	1028.000000	1068.000000

**Fig. 5:**
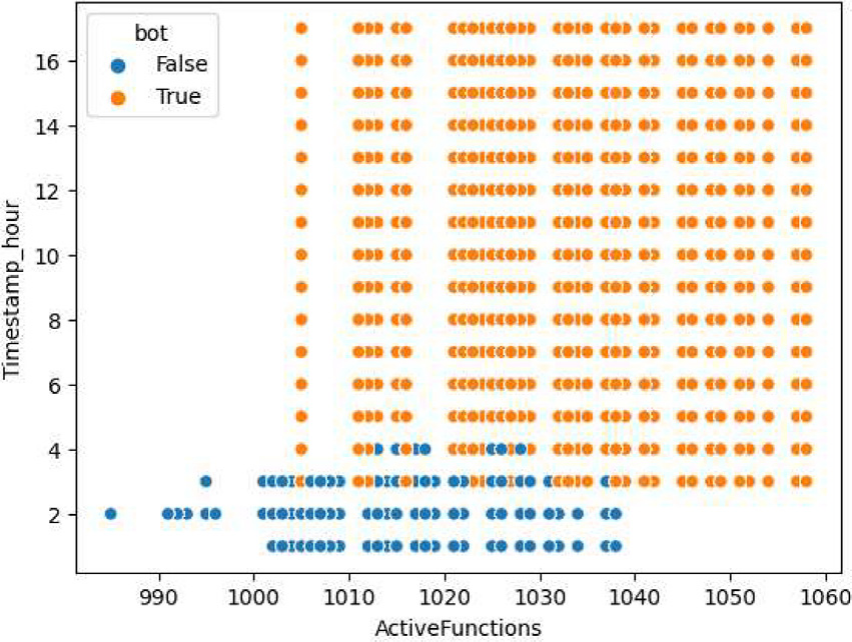
ActiveFunctions vs timestamp_hour.

Fig. 5 shows the relationship between the number of active functions per time interval when the requests are legitimate (bot=0) and when they come from a botnet (bot=1).

**Fig. 6:**
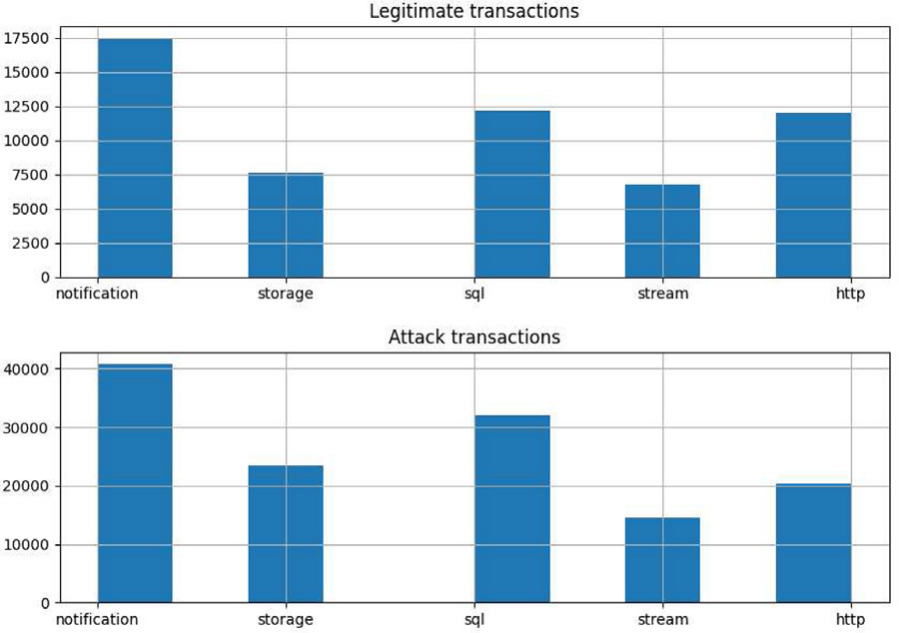
Legitimate vs attack transactions

Finally, we could obtain statistics related to the number of legitimate requests and requests originating from a botnet, grouped by type of event. As illustrated in Fig. 6, we can see that notifications are the most repeated event types.

In a serverless architecture, functions are executed in isolation and on demand in an execution environment managed by the cloud service provider. To generate a dataset that can be used for DoW attack detection, we first simulated function calls in a serverless architecture and combined the results with other datasets containing information related to the number of function calls, dispatch and response times, as well as memory and CPU usage.

This dataset can provide valuable insights and benefits across various aspects of serverless architecture research like performance optimization, resource allocation and cost management. For example, researchers can create Machine learning models to predict future costs and computational resources based on historical data.

The dataset generation process has provided valuable insights and key findings that are instrumental in strengthening our understanding of potential Denial of Wallet (DoW) attacks in serverless architectures. By collecting and analyzing historical data on active functions and resource usage, we have identified important indicators that can help predict and mitigate these financial threats.

Future research can focus on improving serverless workload behavioral analysis. This could include studying user and application behavior patterns to detect anomalies that may lead to DoW attacks. Applying machine learning and deep learning techniques to behavioral analysis can make it more sophisticated, improving the identification of these types of threats.

## Limitations

Not applicable

## Ethics Statement

As we can see in the Rights and permissions section, the code corresponding to the Denial of wallet Test Simulator tool is released in the GitHub repository with a Creative Commons license [12]

The authors confirm that the provided data set and presented work strictly meet the ethics requirements for publication in Data in Brief as mentioned in https://www.elsevier.com/authors/journal-authors/policies-and-ethics

Also indicate that no experiments have been carried out on humans or animals and no data from social networks has been collected.

## CRediT authorship contribution statement

**José Manuel Ortega Candel:** Conceptualization, Methodology, Software, Visualization, Investigation. **Francisco José Mora Gimeno:** Supervision, Validation. **Higinio Mora Mora:** Supervision, Validation.

## Data Availability

Generation of a dataset for DoW attack detection in serverless architectures (Original data) (Mendeley Data)
